# Relative Importance of Climate Variables to Population Vital Rates: A Quantitative Synthesis for the Lesser Prairie-Chicken

**DOI:** 10.1371/journal.pone.0163585

**Published:** 2016-09-29

**Authors:** Julia E. Earl, Samuel D. Fuhlendorf

**Affiliations:** Department of Natural Resource Ecology and Management, Oklahoma State University, Stillwater, Oklahoma, United States of America; Weyerhaeuser Company, UNITED STATES

## Abstract

Climate change is expected to affect temperature and precipitation means and extremes, which can affect population vital rates. With the added complexity of accounting for both means and extremes, it is important to understand whether one aspect is sufficient to predict a particular vital rate or if both are necessary. To compare the predictive ability of climate means and extremes with geographic, individual, and habitat variables, we performed a quantitative synthesis on the vital rates of lesser prairie-chickens (*Tympanuchus pallidictinus*) across their geographic range. We used an information theoretic approach to rank models predicting vital rates. We were able to rank climate models for three vital rates: clutch size, nest success, and subadult/adult seasonal survival. Of these three vital rates, a climate model was never the best predictor even when accounting for potentially different relationships between climate variables and vital rates between different ecoregions. Clutch size and nest success were both influenced by nesting attempt with larger clutches and greater success for first nesting attempts than second nesting attempts. Clutch size also increased with latitude for first nesting attempts but decreased with latitude for second nesting attempts. This resulted in similar clutch sizes for first and second nest attempts at southern latitudes but larger clutches for first nest attempts than second nest attempts at northern latitudes. Survival was greater for subadults than adults, but there were few estimates of subadult survival for comparison. Our results show that individual characteristics and geographic variables are better for predicting vital rates than climate variables. This may due to low samples sizes, which restricted our statistical power, or lack of precision in climate estimates relative to microclimates actually experienced by individuals. Alternatively, relationships between climate variables and vital rates may be constrained by time lags or local adaptation.

## Introduction

Climate change is having large effects on populations, communities, and ecosystems [1, 2]. These effects are expected to continue into the future and increase in magnitude, involving both direct and indirect mechanisms [3, 4]. Populations, particularly, are expected to be affected by climate change through the alteration of vital rates [5], leading to changes in population cycles (e.g., [6]), declines (e.g., [7]), and even extinction [8]. These changes are of great concern for biodiversity conservation [1] and the maintenance of the current level of ecosystems services [9].

Climate change is expected to alter temperature and precipitation averages and variability and the frequency and magnitude of extreme events [10, 11]. Extreme events are characterized as events that are outside the 90^th^ percentile of current climatic events [12]. Events include large storms like tornadoes and hurricanes but also heat waves, extremely cold periods, dry spells, and heavy rain. Extreme events can have large effects on populations [13] and can even drive geographic range shifts [14, 15]. For example, heat waves can cause catastrophic mortality in desert birds [16], whereas extreme ice events can have similar effects on reindeer [17].

Predictions for climate change are complicated by influences on the mean, variability, and extremes of both temperature and precipitation, so it is critical that population ecologists have a better understanding of the relative importance of these factors to species. Understanding the dominant factors driving population vital rates will be critical for understanding population dynamics and developing efficient conservation frameworks. Several recent empirical and theoretical studies have examined the relative importance of climate means and variability to vital rates and population dynamics (e.g., [18, 19, 20]). However, similar evaluations involving climate extremes are still lacking. With an increasing recognition of the likely increase in climate extremes [12], population biologists need to assess the importance of climate extremes to vital rates [21, 22].

Lesser prairie-chickens (*Tympanuchus pallidicinctus*) are a lekking species of conservation concern found in the southern plains of New Mexico, Texas, Oklahoma, Kansas, and Colorado. Lesser prairie-chickens are one species thought to be vulnerable to changes in climate due to their sensitivity to drought [23], their already vulnerable population status [24], and the predicted future climate change across their geographic distribution [10]. In the Southern High Plains of Texas and New Mexico, higher temperatures are expected to reduce lesser prairie-chicken nest survival through relationships with drought and temperatures exceeding the temperature threshold for egg viability [23]. Climate variables are also likely to affect lesser prairie-chicken vital rates through changes in vegetation, and prey and surface water availability with implications for bird physiology, behavior, and stress, which are all relevant to survival and potentially clutch size and nest initiation probability. These changes in nesting females may also alter nest attendance and nesting habitat selection with repercussions for eggs and chicks.

To compare the relative importance of climate means and extremes, we performed a quantitative synthesis of the vital rates of lesser prairie-chickens using an information-theoretic approach [25] that weighted vital rate estimates by their sample size. For vital rates with larger numbers of samples (N > 20; clutch size, nest success, subadult/adult survival), we took a two-step approach in which we first assessed the relative impact of factors known to affect vital rates, such as habitat, latitude, and individual characteristics by ranking models with AIC. We then used the best-fit model as a null model for comparison with climate models. We compared eleven climate models: null, average temperature, average precipitation, extreme temperature, extreme precipitation, drought, and the interactions between each climate variable and ecoregion (a group of five additional models). Models containing interactions between ecoregion and climate variables allowed us to assess the possibility that the effect of climate variables on vital rates may be different in different portions of the lesser prairie-chicken geographic range. We additionally report summarized estimates for all vital rates with lower sample sizes, including egg hatchability, chick survival, and probabilities of nest initiation, nest re-initiation, and brood success.

## Materials and Methods

We located articles by searching Google Scholar, Web of Science, Biological Abstracts, BioOne Abstracts, and Ebsco Wildlife and Ecology Studies Worldwide using the search terms “lesser prairie-chicken” and “nest success,” “survival,” or “clutch.” We completed the final search on 10 December 2014 and included 25 papers in the analysis (Table 1). We included all published journal articles, book chapters, and theses found in this search that met our eligibility criteria, described as follows. Papers included in the quantitative synthesis needed to report a lesser prairie-chicken vital rate and the associated sample size, study site location, year(s) of data collection, and the method for data collection. Studies were excluded (listed in S1 File) if the data was repeated in multiple studies (e.g., [23, 26, 27–31]), the information available was insufficient for a quantitative synthesis [32], the estimates combined data for both lesser and greater prairie-chickens [33], or information was combined for non-adjacent sites (e.g., New Mexico and Oklahoma [34]). We examined all studies that included data from the same study site during the same time period and eliminated any estimates that could include the same individuals or the same nests. For these cases, we chose to use the data that was most specific, such as using vital rate estimates separated for each year rather than summarized for multiple years. For each study, we extracted data on the following demographic rates: nest initiation probability, nest re-initiation probability (given failure of first nest), nest success (the probability that at least one egg hatches in a nest), egg hatchability (the probability that an egg hatches), clutch size, brood success (the probability that at least one chick in a brood survives a given time period), and seasonal survival for both sexes. For clutch size and nest success, we recorded whether estimates were for first nest attempts, second nest attempts, or a combination of first and second nest attempts. We additionally recorded the study site location, the year(s) of data collection, the time of year data was collected, the sex and age of the individuals, the sample size, the habitat type (sand sagebrush, sand shinnery oak, or a combination), and the estimation method for the vital rate. For almost all vital rates, estimates were made by monitoring individuals with radiotransmitters attached. Survival estimates using radiotransmitters appear relatively unbiased [30]. However, radiotransmitter estimates could bias estimates of nest initiation, nest reinitiation, or nest success if some nests failed before they were detected by researchers. During data extraction, two people assessed and recorded data from each paper independently. Where discrepancies occurred, discussion and rereading of text was used to generate consensus.

**Table 1:** Studies included in quantitative synthesis of lesser prairie-chicken vital rates (note that additional vital rates may have been published in a study that was not included due to overlap with another study).

Study	Years	Location	Vital Rates
Boal et al. 2010 [35]	2008, 2009	Cochran, Hockley, Terry, Yoakum Co., TX	Nest initiation, nest success
Campbell 1972 [36]	1962–1970	Roosevelt, Lea Co., NM	Subadult/adult survival
Copelin 1963 [37]	1959	Ellis Co., OK	Clutch size, hatchability, nest success
Davis 2009 [38]	2004–2005	Roosevelt Co., NM	Brood success, nest initiation, nest success, renest initiation
Fields 2004 [39]	2002–2004	Gove Co., KS	Chick survival, clutch size, hatchability, nest initiation, nest success, renest initiation
Grisham 2012 [40]	2001–2010	Roosevelt Co., NM; Cochran, Hockley, Terry, Yoakum Co., TX	Brood success, subadult/adult survival, nest success
Grisham et al 2014 [41]	2008–2011	Cochran, Hockley, Terry, Yoakum Co., TX	Clutch size, hatchability, nest initiation, nest success, renest initiation
Hagen et al. 2002 [42]	1997–1999	Finney Co., KS	Nest success
Hagen et al. 2007 [43]	1998–2002	Finney Co., KS	Subadult/adult survival
Holt 2012 [44]	2008–2010	Gray, Hemphill Co., TX	Clutch size, subadult/adult survival, nest success
Jamison 2000 [45]	1997–1999	Finney Co., KS	Subadult/adult survival
Jones 2009 [46]	2001–2003	Hemphill, Lipscomb, Wheeler Co., TX	Subadult/adult survival, nest success
Kukal 2010 [47]	2008–2010	Gray, Hemphill Co., TX	Subadult/adult survival
Leonard 2008 [48]	2006–2007	Cochran, Yoakum Co., TX	Subadult/adult survival, nest success, renest initiation
Lyons et al. 2009 [49]	2003–2005	Cochran, Yoakum Co., TX	Subadult/adult survival
Lyons et al. 2011 [50]	2001–2007	Hemphill, Wheeler, Lipscomb Co., Cochran, Yoakum Co., TX	Nest initiation, renest initiation
Merchant 1982 [51]	1979–1980	Lea, Roosevelt, Co., NM	Clutch size, nest initiation, nest success, renest initiation
Patten et al. 2005 [52]	1999–2003	Beaver, Ellis, Harper Co., OK; Roosevelt Co., NM	Clutch size
Pirius et al. 2013 [53]	2008–2011	Cochran, Hockley, Terry, Yoakum Co., TX	Subadult/adult survival
Pitman 2003 [54]	1998–2002	Finney Co., KS	Chick survival
Pitman et al. 2005 [55]	1997–2002	Finney Co., KS	Nest success
Pitman et al. 2006 [56]	1997–2002	Finney Co., KS	Clutch size, nest initiation, renest initiation
Pitman et al. 2006 [57]	1997–2003	Finney Co., KS	Brood success
Riley et al. 1992 [58]	1976–1978	Chaves Co., NM	Nest success
Toole 2005 [59]	2001–2002	Hemphill, Lipscomb, Wheeler Co., TX	Subadult/adult survival

We obtained monthly climate data from the National Oceanic and Atmospheric Administration (NOAA) for each year and site that we had vital rate estimates. Most site-level data were reported by county or a group of counties, so we obtained climate data spanning the appropriate years from a weather station closest to the geographic center of the county or counties. Data was checked for completeness and summarized for each demographic rate. We excluded any data from any month with more than 5 days of missing data. For clutch size, we examined the weather during the previous six months (October to March). For nest success, we examined weather during the nesting period (April, May, June). We analyzed chick survival separately from subadults and adults. We examined the daily survival rate for chicks and seasonal survival (i.e., all survival estimates converted to that for a three-month period) for subadults and adults and used weather data for the period survival was estimated. Subadult/adult survival was categorized by season into warm seasons for spring and summer (March to August) and cool seasons for fall and winter (September to February). For vital rate estimates from studies that averaged over multiple years, we used the median year and mean climate variables in the models.

We classified climate data into four groups: extreme temperature, average temperature, extreme precipitation, and average precipitation. Extreme temperature variables included the number of hot days (maximum temperature over 32.2°C), number of freezing days (maximum temperature below freezing), number of extremely cold nights (minimum temperature below -17.8°C), the extreme maximum temperature, and the extreme minimum temperature. Average temperature variables included the number of freezing nights, the mean daily maximum temperature, the mean daily minimum temperature, and the mean daily temperature. Extreme precipitation variables included the number of days with heavy rain (daily precipitation greater than 2.5 cm), the maximum daily precipitation, and the maximum snow depth. Average precipitation variables included the number of rainy days (precipitation greater than 0.25 cm), the total precipitation, and the total snowfall. For each data set, we first assessed the correlations among climatic variables within each group. For correlated variables (|r|> 0.7), we kept the variable most highly correlated with the vital rate of interest. For an indication of drought, we used the Palmer Drought Severity Index, where index values -4.0 or below indicate extreme drought and index values +4.0 or above indicate extreme wet conditions [60]. We used historical drought data from NOAA calculated for multi-county US Climate regions in each state. Our data covered two climate regions in Kansas (regions 4 and 7), one in Oklahoma (region 1), two in New Mexico (regions 3 and 7), and two in Texas (regions 1 and 2). For cases where a study site spanned two climatic regions, we calculated the average of the two regions for the time period.

For the analyses, we used the vital rate estimate as a response variable in general linear models. Note that because of the nature of the study, the data does not lend itself to calculating traditional effects sizes. As such, there is no established method for assessing publication bias. However, there is also less concern about potential bias relative to studies calculating traditional effect sizes, because there are not non-significant results that might be less likely to be published, and we also included graduate theses. One potential source of bias is a change in the established methods for estimating vital rates through time. To potentially account for this, we included a model with the year(s) that each vital rate was estimated as a predictor variable (see below).

For each analysis, we used one vital rate estimate as a replicate. Each replicate was weighted by the sample size to account for differences in precision among vital rate estimates [61]. We attempted to include the study as a random variable to account for possible non-independence between estimates made by the same researchers, but a strong relationship between study and climate variables prevented the inclusion of both factors in the models. As such, analyses did not include any random variables. We partitioned vital rates into three categories based on sample size and performed analyses accordingly. For vital rates with less than ten estimates, we report the weighted mean. For vital rates with 10–20 estimates, we restricted the model set to seven models (year, latitude, ecoregion, individual, habitat, nesting attempt [for nest success and clutch size]; null [intercept-only]; Table 2), which we will refer to as the limited model set. For ecoregion, we established two categories of ecoregion based on the four ecoregions specified in McDonald et al. [62]. The two categories were the sand shinnery oak prairie ecoregion (including New Mexico and the southern portion of the Texas pandhandle) and all other ecoregions (including northern Texas, Oklahoma, Kansas, and Colorado). These two areas are spatially separate portions of the lesser prairie-chicken geographic distribution, and there is evidence that there is no genetic or demographic connectivity between the two areas [63, 64]. We also included a time model for chick survival that included a parameter for the number of days over which survival was estimated. We further included post hoc models for clutch size containing the interaction between nesting attempt and other key variables, including year, latitude, ecoregion, age class, and habitat. For vital rates with greater than 20 estimates, we first ranked models in the limited model set (Table 2). We included variables with significant parameter estimates (i.e. 95% confidence intervals that did not include zero) found in the best and competing models in all of the climate models. We examined eleven climate models: null, drought, average temperature, average precipitation, extreme temperature, extreme precipitation, and five additional models with the interaction between each of the five types of climate variables and ecoregion (Table 2). We wanted to include the ecoregion-climate variable interaction models to account for the possibility that climate variables affected vital rates differently in the two different portions of the lesser prairie-chicken’s geographic distribution. For all climate models, we checked the variance inflation factors for all variables to determine the degree of multicollinearity. Variables in all models were included as fixed effects and had variance inflation factors less than four. Models were ranked using Akaike’s Information Criterion corrected for small samples sizes (AIC_c,_), where the lowest value is considered to be the best model in the set of models and models within two units are competing models [65, 66]. We also computed Akaike weights to evaluate the relative support for each model. In cases with competing models, we used model averaging to make parameter estimates. All models were implemented in R [67] using standard functions and the AICcmodavg package. Nest initiation, chick survival, and adult/subadult survival were arcsine transformed, renest initiation was square root transformed, and nest success was log transformed to meet assumptions of normality and homoscedasticity.

**Table 2:** Model sets used in the analysis of lesser prairie-chicken vital rates.

Model Set	Model	K	Parameters
Limited Model Set	Null	2	Intercept only
	Habitat	4	Habitat (sand sagebrush, sand shinnery oak, mixed)
	Year	3, 5	Year of study; season^1^ (warm, cool, both)
	Individual	4, 6	Sex^1^ (male, female, both); age class (subadult, adult, both)
	Latitude	3	Latitude of study site
	Ecoregion	3	Ecoregion (sand shinnery oak prairie, other ecoregion)
	Nesting attempt^2^	4	Nesting attempt (1^st^, 2^nd^, both)
	Nesting attempt interactions (set including 5 models)^3^	7, 10	Nesting attempt (1^st^, 2^nd^, both); other variables (year, latitude, ecoregion, habitat, or age class); interaction terms
	Time^4^	3	Number of days included in original estimate
Climate models	Null	4–7	Parameters from best model in limited model set (also included in all climate models)
	Average Temperature	6–9	Mean maximum daily temperature^5^; mean minimum daily temperature^2^; number of days with a minimum below freezing^6^
	Average Precipitation	6–9	Total precipitation^7^; total snow fall; number of days with more than 0.25 cm of precipitation^5^
	Extreme Temperature	6–9	Number of days with a high over 32.2°C^5^; extreme maximum temperature^7^; extreme minimum temperature^2^; number of days with a minimum below -17.8°C^1^
	Extreme Precipitation	6–9	Number of days with greater than 2.5 cm of precipitation; maximum snow depth
	Drought	5–8	Palmer drought severity index
	Climate interaction with ecoregion (set of 5 models)	7–12	Ecoregion; climate variable(s); interaction between ecoregion and climate variables

^1^ Only included in the analysis of subadult/adult survival.

^2^ Only included in the analysis of clutch size and nest success.

^3^ Only included in the analysis of clutch size; developed as post-hoc models.

^4^ Only included in the analysis of subadult/adult and chick survival.

^5^ Only included in the analysis of clutch size and subadult/adult survival.

^6^ Only included in the analysis of nest success and subadult/adult survival.

^7^ Only included in the analysis of nest success.

## Results

We found 44 relevant papers of which 25 contained sufficient data for the quantitative synthesis and met all of our eligibility requirements. Studies were conducted across the lesser prairie-chicken geographic range, except for Colorado (Table 1, data in S2 File). Egg hatchability and brood success had low sample sizes, and as such, we only present the weighted means (± standard error). Egg hatchability was estimated as 0.918 ± 0.043 (N = 5 estimates, 125 total nests) at three sites in Kansas, Oklahoma, and Texas, which included all three habitat types. Brood success was estimated at three sites in Kansas, New Mexico, and Texas, which also included all three habitat types. Brood success was estimated for time periods from 18 to 68 days, but the amount of time examined did not affect the estimate (p = 0.86). Brood success was estimated as 0.134 ± 0.072 (N = 5 estimates, 246 total broods).

**Fig 1:**
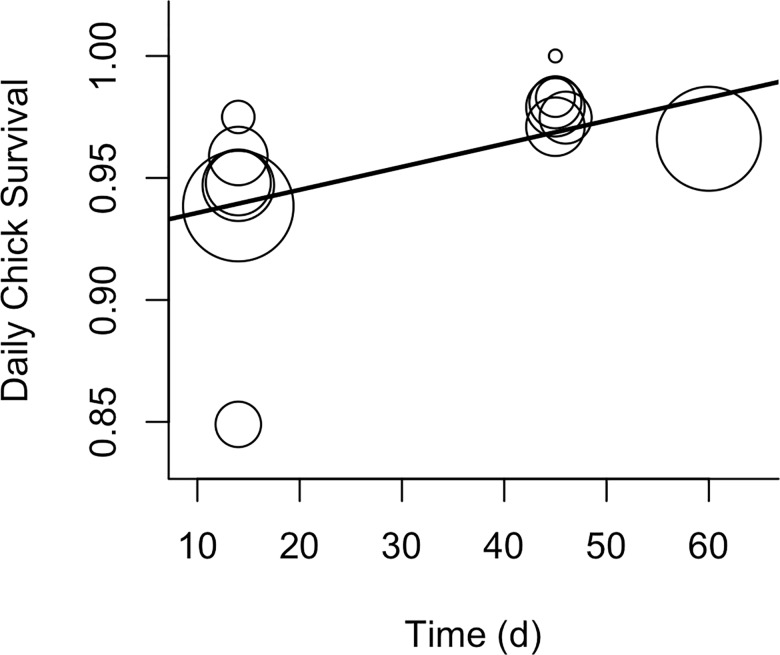
Relationship between lesser prairie-chicken daily chick survival and the amount of time over which survival was estimated. Circles indicate the relative sample size for each survival estimate.

Nest initiation, nest re-initiation, and chick survival had intermediate sample sizes. As such, we used the limited model set. The nest initiation probability was estimated at two sites in Kansas and Texas, and one site in New Mexico. The latitude model was the best model with the null and habitat models competing (Table 3; S1 Table for full AIC tables). However, only habitat parameters had confidence intervals that did not include zero. The nest initiation probability (N = 13 estimates, 370 females) was lower in mixed habitat (0.6381 ± 0.102) than sand sagebrush (0.936 ± 0.062) with sand shinnery oak intermediate (0.787 ± 0.112). Nest re-initiation was also estimated at two sites in Kansas and Texas, and one site in New Mexico. The best model for nest re-initiation was the ecoregion model with the null model and latitude model competing (Table 3 and S1 Table). However, model-averaging revealed that only the intercept had a confidence interval that did not include zero. Nest re-initiation was estimated at 0.213 ± 0.050 (N = 12 estimates, 204 females). Chick survival was estimated for only two sites in Kansas (both sand sagebrush; N = 13 estimates, 117 chicks). Because of the lack of variability in many of the parameters (habitat, latitude, sex), the model set for chick survival included only three models: the null, year, and time. The time model was the best (r^2^ = 0.36) with the null model competing (Table 3 and S1 Table). Daily chick survival increased with the number of days that survival was estimated [arcsine(survival) = 1.190 (± 0.045) + 0.003(± 0.0012)*days], where survival estimates were made over 14 to 60 days (Fig 1).

**Table 3:** Model ranking for the limited model set for each vital rate, where K is the number of parameters and *ω*_*i*_ is the model weight.

Vital Rate	Model	K	ΔAIC	*ω*_*i*_
Nest Initiation	Latitude	3	0	0.34
	Null	2	0.36	0.29
	Habitat	4	1.95	0.13
	Ecoregion	3	2.04	0.12
	Year	3	3.45	0.06
	Individual	4	3.50	0.06
Nest Re-initiation Rate	Ecoregion	3	0	0.39
	Null	2	0.73	0.27
	Latitude	3	0.90	0.25
	Year	3	3.95	0.05
	Individual	4	4.91	0.03
	Habitat	4	10.91	< 0.01
Chick Survival	Time	3	0	0.74
	Null	2	2.39	0.22
	Year	3	5.72	0.04
Clutch Size	Nesting attempt, latitude interaction	7	0	0.99
	Nesting attempt, ecoregion interaction	7	8.97	0.01
	Nesting attempt, year interaction	7	33.39	< 0.01
	Nesting attempt habitat interaction	10	34.90	< 0.01
	Nesting attempt	4	35.82	< 0.01
	Ecoregion	3	44.85	< 0.01
	Latitude	3	45.62	< 0.01
	Nesting attempt, age class interaction	7	47.81	< 0.01
	Habitat	4	53.20	< 0.01
	Null	2	60.44	< 0.01
	Individual	4	60.74	< 0.01
	Year	3	62.40	< 0.01
Nest Success	Nesting attempt	4	0	0.93
	Null	2	6.65	0.03
	Year	3	9.06	0.01
	Ecoregion	3	9.06	0.01
	Latitude	3	9.07	0.01
	Habitat	4	10.4	0.01
	Individual	4	11.22	< 0.01
Subadult/Adult Survival	Individual	6	0	0.98
	Habitat	4	8.75	0.01
	Null	2	12.02	< 0.01
	Year	6	12.07	< 0.01
	Latitude	3	14.01	< 0.01
	Ecoregion	3	14.37	< 0.01

**Fig 2:**
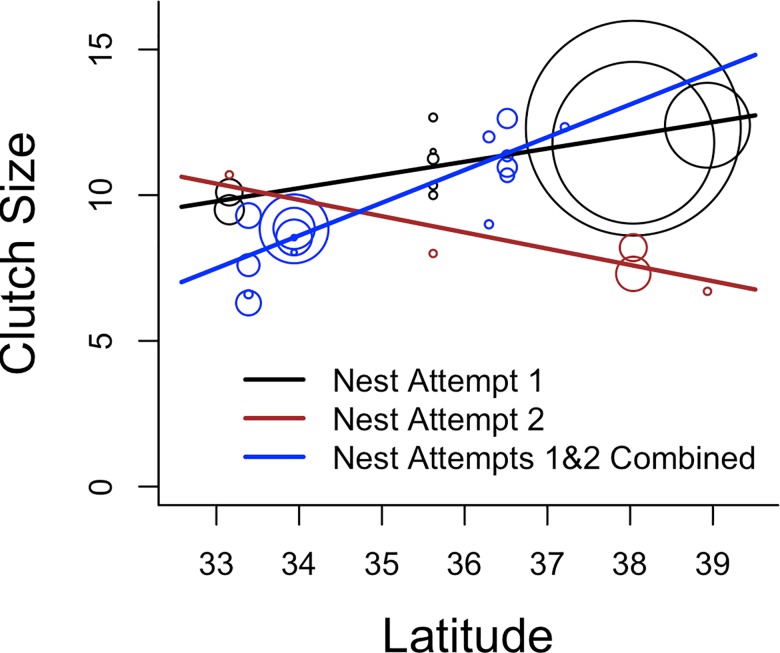
Relationship between lesser prairie-chicken clutch size and latitude of the population. Circles indicate the relative sample size for each clutch size estimate.

We examined climate models for clutch size, nest success, and subadult/adult seasonal survival (i.e. for three-month periods). Clutch size was estimated in all four states (N = 31 estimates, 363 nests), including three sites in Kansas and two sites each in New Mexico, Oklahoma, and Texas. The best model from the limited model set was the nesting attempt latitude interaction model with a high model weight (0.99; Tables 3 and S1). As such, nesting attempt, latitude, and the interaction were used as the null model, which was the best out of the climate model set with no models competing (Tables 4 and S2). Clutch size increased with latitude for first nesting attempts, but decreased with latitude for second nesting attempts (r^2^ = 0.91; Fig 2). This relationship resulted in very little difference in clutch size between nesting attempts in the southern latitudes, but first nesting attempts had greater clutch sizes than second nesting attempts in the northern latitudes.

**Table 4:** Model ranking for climate models for each vital rate, where parameters included in all models for each vita rate are in parentheses, K is the number of parameters, and *ω*_*i*_ is the model weight.

Vital Rate	Model	K	ΔAIC_c_	*ω*_*i*_
Clutch Size	Null (nest attempt, latitude interaction)	7	0	0.54
	Average precipitation, ecoregion interaction	10	2.01	0.20
	Drought	8	2.74	0.17
	Extreme precipitation	9	4.85	0.06
	Extreme temperature	9	5.92	0.03
	Average temperature	9	5.94	0.03
	Average precipitation	9	6.20	0.03
	Drought, ecoregion interaction	10	10.34	< 0.01
	Average temperature, ecoregion interaction	12	14.52	< 0.01
	Extreme precipitation, ecoregion interaction	12	14.59	< 0.01
	Extreme temperature, ecoregion interaction	12	20.58	< 0.01
Nest Success	Null (nest attempt)	4	0	0.46
	Drought	5	1.91	0.18
	Extreme temperature	6	2.74	0.12
	Extreme precipitation	6	3.33	0.09
	Average precipitation	6	3.47	0.08
	Average temperature	5	4.21	0.06
	Drought, ecoregion interaction	7	5.70	0.02
	Average precipitation, ecoregion interaction	9	12.49	< 0.01
	Extreme precipitation, ecoregion interaction	9	12.53	< 0.01
	Extreme temperature, ecoregion interaction	9	13.09	< 0.01
	Average temperature, ecoregion interaction	9	13.38	< 0.01
Subadult/Adult Survival	Null (age)	4	0	0.27
	Extreme Precipitation	6	0.01	0.27
	Average Precipitation	6	1.65	0.12
	Average Temperature	6	1.69	0.12
	Drought	5	1.79	0.11
	Extreme Temperature	6	1.94	0.10
	Drought, ecoregion interaction	7	7.65	0.01
	Extreme temperature, ecoregion interaction	9	7.69	0.01
	Extreme precipitation, ecoregion interaction	9	10.49	< 0.01
	Average temperature, ecoregion interaction	9	10.61	< 0.01
	Average precipitation, ecoregion interaction	9	11.85	< 0.01

Nest success was estimated in all four states (N = 32 estimates, 506 nests), including four sites in Texas, three sites in New Mexico, two sites in Kansas, and only one site in Oklahoma. The best model from the limited model set was the nesting attempt with a model weight of 0.93 (Table 3). We included nesting attempt in all of the climate models. Of the climate model set, the null model containing only nesting attempt was the best (model weight = 0.46; Table 4) with the drought model competing. However, the drought parameter had a confidence interval that overlapped zero. Second nesting attempts (0.091 ± 0.245) had lower nest success than first nesting attempts (0.632 ± 0.091), and estimates that included both first and second nesting attempts were intermediate (0.297 ± 0.091).

Seasonal subadult/adult survival was estimated at one site in Kansas, four sites in Texas, and one site in New Mexico (N = 32 estimates with a combined samples size of 1395 individuals). The best model from the limited model set was the individual model with no models competing (Table 3). Only the age class parameters had confidence intervals that did not include zero; these were incorporated into all climate models. This null model was the best climate model (r^2^ = 0.44, Table 4) with several climate models competing. However, all of the climate variable slopes had confidence intervals that included zero. Seasonal survival (i.e. over three months) was greater for mixed age classes (0.902 ± 0.035) than adults (0.733 ± 0.027) with intermediate survival for subadults (0.834 ± 0.063).

## Discussion

We were able to compare the importance of climate means and extremes for three lesser prairie-chicken vital rates: clutch size, nest success, and subadult/adult seasonal survival. Climatic factors were never included in the best model. Previous work has examined climate extremes and averages separately, and it is clear that both can affect population vital rates (e.g., [16, 19]). Sometimes temperature averages and extremes can affect the same vital rate simultaneously, as was found for the greater sage-grouse, *Centrocercus urophasianus* [68], and recent reviews have highlighted the need to include extreme events in climate change experiments on communities and ecosystems [21, 22]. However, our results show that life history and geographic factors can be better predictors of vital rates than climate variables in some cases, though this result may be due to methodology.

The lack of importance of climate variables may have occurred for a number of reasons. First, our sample size may have been too low to account for relationships with climate variables, and our method of accounting for vital rate precision with sample size may have had low accuracy. Second, climate data from nearby weather stations may not accurately reflect the microclimates actually experienced by individuals. Different microhabitats are known to greatly affect the microclimates and operative temperatures experienced by individuals [69], and lesser prairie-chickens select microclimates that are cooler and more humid, which facilitates their survival [32] and is dependent on relative conditions [70]. If broader weather station data does not accurately reflect microclimates experienced by individuals, syntheses combining published data with off-study site climate data may have only a limited ability to detect trends. However, our results showing a lack of trends between climate and vital rates may also be due to time lags in climate variable effects or result from adaptations to different climatic regimes in different areas of the lesser prairie-chicken geographic distribution.

Previous work has found that climatic variables can affect lesser prairie-chicken vital rates, particularly in the Southern High Plains. For example, increases in winter temperature decrease daily nest survival [23]. There is also evidence that lesser prairie-chicken nest temperatures can exceed the threshold for egg viability during extreme heat waves [23]. These types of relationships may be different in other parts of the geographic distribution or may be stronger with different climate variables. While we did examine different relationships between climate variables and vital rates in different areas of the geographic range, our statistical power to evaluate these more complex relationships was limited.

Clutch size in temperate birds typically increases with latitude [71, 72]. Our data also shows this trend for first nesting attempts, which has been suggested previously for lesser prairie-chickens [52]. However, clutch size decreased with latitude for second nesting attempts, a trend that was based on a much smaller sample size than the first nesting attempt. This results in similar clutch sizes for first and second nest attempts at the southern end of the distribution, but much greater clutch sizes for first nest attempts than second nest attempts at the northern end of the distribution. Latitude integrates a number of climatic and habitat factors that may cause it to have a greater predictive power than any of those variables by themselves. However, previous work has found weak or no relationship between clutch size and climate in lesser prairie-chickens [23, 52], which is consistent with our results. Also, latitudinal changes in climate and habitat likely lead to greater fitness payoffs for different reproductive strategies along that gradient. Across many bird species, there is a tradeoff between clutch size and the number of nesting attempts that corresponds with latitude [71], which may be driven by latitudinal changes in the onset and duration of the breeding season [73]. Although the latitude model was a competing model for the nest re-intiation rate, the parameter estimate’s confidence interval included zero, which is not consistent with a tradeoff between clutch size and nest re-intiation rate. Some birds also trade off more clutches with fewer eggs per clutch in the face of higher predation risk as a bet-hedging strategy [74].

Lesser prairie-chicken nest success was greater for first nesting attempts than second nesting attempts. This is contrary to the closely related greater prairie-chicken (*Tympanuchus cupido*), which has greater nest success for second nesting attempts [75]. Additionally, sage-grouse display no difference in nest success between first and second nesting attempts [76]. Further modeling work will be necessary to understand how these changes affect recruitment and population growth rates across the lesser prairie-chicken geographic range.

Our modeling of lesser prairie-chicken subadult/adult seasonal survival (i.e. over three months) showed no effect of any climate variables. Because of the limited sample size and the vast number of different periods of time over which survival was estimated, our power to detect effects of climate was likely limited. Standardized time periods would likely increase the ability to detect overall trends in future studies. We did find that subadults had greater survival than adults overall. However, there were few estimates of subadult survival when compared with adult survival in the data set, and this likely skewed this result. A greater number of subadult survival estimates will be necessary to assess this trend.

Our results show that life history and geography are the primary factors affecting lesser prairie-chicken vital rates. We found no effects of climate. Other studies have found equal importance of climate averages and extremes [68], but few studies have compared climatic averages and extremes directly. Including both averages and extremes greatly complicates models predicting the current and future effects of climate change on population dynamics. As such, there is great value in performing additional studies comparing climate averages and extremes across a variety of species. With greater information, we can determine whether only one type of climate variable can result in robust population predictions or whether both or none are necessary. Expanding this type of work will help determine which species are most vulnerable to a changing climate and how managers may be able to mitigate that change.

## Supporting Information

S1 File List of reasons for excluding studies from quantitative synthesis. (DOCX)

S2 File Data used in quantitative synthesis of lesser prairie-chicken vital rates. (CSV)

S3 File PRISMA checklist showing compliance to standards for meta-analysis and quantitative reviews for the quantitative synthesis of lesser prairie-chicken vital rates. (DOC)

S4 File PRISMA flow chart showing steps of gathering studies and assessing whether they met inclusion criteria for the quantitative synthesis of lesser prairie-chicken vital rates. (DOCX)

S1 Table AIC_c_ tables for the limited model set predicting variability in lesser prairie-chicken vital rates, where K is the number of parameters and N is the sample size (number of estimates). (DOCX)

S2 Table AIC_c_ tables for the climate model set predicting variability in lesser prairie-chicken vital rates, where K is the number of parameters and N is the sample size (number of estimates). (DOCX)
