# Supplementary material for: Relative Importance of Climate Variables to Population Vital Rates: A Quantitative Synthesis for the Lesser Prairie-Chicken
Source: PLoS One. 2016 Sep 29;11(9):e0163585. doi: 10.1371/journal.pone.0163585 (PMC5042413; doi:10.1371/journal.pone.0163585)
Supplement: S1 File — (DOCX) [file pone.0163585.s001.docx]

Appendix A. Reasons for excluding studies from quantitative synthesis.

Bell et al. (2007): No vital rate information.

Copelin (1959): No vital rate information.

Crawford (1974): No vital rate information.

Davis et al. (1979): Information repeated in other studies.

Fields et al. (2006): Information combines lesser and greater prairie chickens.

Grisham et al. (2013): Information repeated in other studies.

Hagen et al. (2004): Overview of all of Hagen’s work, no new vital rate information.

Hagen et al. (2005): Information repeated in other studies.

Hagen et al. (2006): Information repeated in other studies.

Johnson et al. (2004): No vital rate information, focuses on vegetation.

Lyons (2008): All information published in other sources.

Oblawsky (1987): No vital rate information.

Patten et al. (2005a): No information on survival, just analysis of factors related to survival.

Pirius (2011): All information published in Pirius et al. (2013).

Pruett et al. (2011): All information published in Patten et al. (2005b).

Robel et al. (2003): Information repeated in other studies.

Schroeder and Baydack (2001): No new vital rate information, review paper.

Wisdom (1980): Information repeated in other studies.

Wolfe et al. (2007): Contained data summarized for two nonadjacent sites from New Mexico and Oklahoma.

**Literature Cited**

Bell, L. A., J. C. Pitman, M. A. Patten, D. H. Wolfe, S. K. Sherrod, and S. D. Fuhlendorf. 2007. Juvenile Lesser Prairie-Chicken growth and development in southeastern New Mexico. Wilson Journal of Ornithology **119**:386-391.

Copelin, F. F. 1959. Notes regarding the history and current status of the lesser prairie-chicken in Oklahoma. Proceedings of the Oklahoma Academy of Science **37**:158-161.

Crawford, J. A. 1974. The Effects of Land Use on Lesser Prairie-Chicken Populations in West Texas. Texas Tech University, Lubbock, TX.

Davis, C. A., T. Z. Riley, H. R. Suminski, and M. J. Wisdom. 1979. Habitat evaluation of Lesser Prairie Chickens in eastern Chaves Co., New Mexico. Final Report to BLM, Roswell, Contract YA-512-CT6-61.*in* B. o. L. Management, editor. New Mexico State University, Las Cruces NM.

Fields, T. L., G. C. White, W. C. Gilgert, and R. D. Rodgers. 2006. Nest and brood survival of Lesser Prairie-Chickens in west central Kansas. Journal of Wildlife Management **70**:931-938.

Grisham, B. A., C. W. Boal, D. A. Haukos, D. M. Davis, K. K. Boydston, C. Dixon, and W. R. Heck. 2013. The predicted influence of climate change on lesser prairie-chicken reproductive parameters. PLoS One **8**:e68225.

Hagen, C. A., B. E. Jamison, K. M. Giesen, and T. Z. Riley. 2004. Guidelines for managing lesser prairie-chicken populations and their habitats. Wildlife Society Bulletin **32**:69-82.

Hagen, C. A., J. C. Pitman, B. K. Sandercock, R. J. Robel, and R. D. Applegate. 2005. Age-specific variation in apparent survival rates of male Lesser Prairie-Chickens. Condor **107**:78-86.

Hagen, C. A., B. K. Sandercock, J. C. Pitman, R. J. Robel, and R. D. Applegate. 2006. Radiotelemetry survival estimates of Lesser Prairie-Chickens in Kansas: Are there transmitter biases? Wildlife Society Bulletin **34**:1064-1069.

Johnson, K., B. H. Smith, G. Sadoti, T. B. Neville, and P. Neville. 2004. Habitat use and nest site selection by nesting lesser prairie-chickens in southeastern New Mexico. Southwestern Naturalist **49**:334-343.

Lyons, E. K. 2008. Lesser prairie-chicken demographics in Texas: survival, reproduction, and population viability. Texas A&M University, College Station, TX.

Oblawsky, C. D. 1987. Effects of Shinnery Oak Control with Tebuthiuron on Lesser Prairie-Chicken Populations. Texas Tech University, Lubbock, TX.

Patten, M. A., D. H. Wolfe, E. Shochat, and S. K. Sherrod. 2005a. Effects of microhabitat and microclimate selection on adult survivorship of the Lesser Prairie-Chicken. Journal of Wildlife Management **69**:1270-1278.

Patten, M. A., D. H. Wolfe, E. Shochat, and S. K. Sherrod. 2005b. Habitat fragmentation, rapid evolution and population persistence. Evolutionary Ecology Research **7**:235-249.

Pirius, N. E. 2011. The non-breeding season ecology of Lesser Prairie-Chickens (*Tympanuchus pallidicinctus*) in the southern high plains of Texas. Texas Tech University, Lubbock.

Pirius, N. E., C. W. Boal, D. A. Haukos, and M. C. Wallace. 2013. Winter habitat use and survival of Lesser Prairie-Chickens in west Texas. Wildlife Society Bulletin **37**:759-765.

Pruett, C. L., J. A. Johnson, L. C. Larsson, D. H. Wolfe, and M. A. Patten. 2011. Low effective population size and survivorship in a grassland grouse. Conservation Genetics **12**:1205-1214.

Robel, R. J., T. L. Walker, Jr., C. A. Hagen, R. K. Ridley, K. E. Kemp, and R. D. Applegate. 2003. Helminth parasites of Lesser Prairie-Chicken *Tympanuchus pallidicinctus* in southwestern Kansas: incidence, burdens and effects. Wildlife Biology **9**:341-349.

Schroeder, M. A., and R. K. Baydack. 2001. Predation and the management of prairie grouse. Wildlife Society Bulletin **29**:24-32.

Wisdom, M. J. 1980. Nesting habitat of Lesser Prairie Chickens in eastern New Mexico. New Mexico State University, Las Cruces.

Wolfe, D. H., M. A. Patten, E. Shochat, C. L. Pruett, and S. K. Sherrod. 2007. Causes and patterns of mortality in Lesser Prairie-chickens *Tympanuchus pallidicinctus* and implications for management. Wildlife Biology **13**:95-104.
