# Supplementary material for: Relative Importance of Climate Variables to Population Vital Rates: A Quantitative Synthesis for the Lesser Prairie-Chicken
Source: PLoS One. 2016 Sep 29;11(9):e0163585. doi: 10.1371/journal.pone.0163585 (PMC5042413; doi:10.1371/journal.pone.0163585)
Supplement: S4 File — (DOCX) [file pone.0163585.s004.docx]

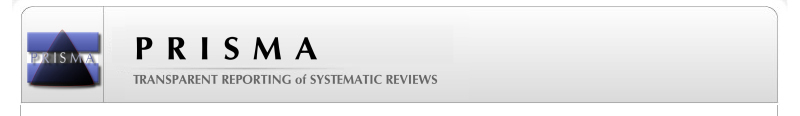
**PRISMA 2009 Flow Diagram**

Records after duplicates and studies not containing relevant information removed
(n = 44 )

Full-text articles excluded for lack of demographic data, duplicate data, insufficient information, combining information on lesser and greater prairie-chickens, and combining information from non-adjacent sites
(n = 19 )

Studies included in quantitative synthesis (meta-analysis)
(n = 25 )

Studies included in qualitative synthesis
(n = 25 )

Full-text articles assessed for eligibility
(n = 44 )

Records excluded
(n = 0 )

Records screened
(n = 44 )

Additional records identified through other sources
(n = 18 )

## Identification

## Eligibility

## Included

## Screening

Records identified through database searching
(n = 96 )
