# Supplementary material for: Relative Importance of Climate Variables to Population Vital Rates: A Quantitative Synthesis for the Lesser Prairie-Chicken
Source: PLoS One. 2016 Sep 29;11(9):e0163585. doi: 10.1371/journal.pone.0163585 (PMC5042413; doi:10.1371/journal.pone.0163585)
Supplement: S1 Table — (DOCX) [file pone.0163585.s005.docx]

Table S1. AIC_c_ tables for the limited model set predicting variability in lesser prairie-chicken vital rates, where K is the number of parameters and N is the sample size (number of estimates).

**Vital Rate Model K Log Likelihood AIC_c_ Δ*_i_* *ω_i_***

Nest Initiation Latitude 3 -3.86 16.39 0 0.34

(N = 13) Null 2 -5.77 16.75 0.36 0.29

Habitat 4 -2.67 18.34 1.95 0.13

Ecoregion 3 -4.88 18.43 2.04 0.12

Year 3 -5.59 19.84 3.45 0.06

Individual 4 -3.44 19.89 3.50 0.06

Nest Re-initiation Rate Ecoregion 3 1.04 6.91 0 0.39

(N =12) Null 2 -1.16 7.65 0.73 0.27

Latitude 3 0.59 7.82 0.90 0.25

Year 3 -0.93 10.86 3.95 0.05

Individual 4 0.94 11.82 4.91 0.03

Habitat 4 1.09 17.82 10.91 < 0.01

Chick Survival Time 3 28.31 -20.15 0 0.74

(N = 13) Null 2 11.48 -17.76 2.39 0.22

Year 3 11.55 -14.43 5.72 0.04

Clutch Size Nesting attempt, latitude 7 -35.28 89.43 0 0.99

(N = 31) interaction

Nesting attempt, ecoregion 7 -39.76 98.39 8.97 0.01

interaction

Nesting attempt, year 7 -51.97 122.81 33.39 < 0.01

interaction

Nesting attempt, habitat 10 -46.66 124.33 34.90 < 0.01

interaction

Nesting attempt 4 -57.86 125.25 35.82 < 0.01

Ecoregion 3 -63.69 134.27 44.85 < 0.01

Latitude 3 -64.08 135.05 45.62 < 0.01

Nesting attempt, individual 7 -57.35 137.24 47.81 < 0.01

interaction

Habitat 4 -66.54 142.63 53.20 < 0.01

Null 2 -72.72 149.86 60.44 < 0.01

Individual 4 -70.32 150.17 60.74 < 0.01

Year 3 -72.47 151.82 62.40 < 0.01

Nest Success Nesting attempt 4 11.35 -13.22 0 0.93

(N = 32) Null 2 5.49 -6.57 6.65 0.03

Year 3 5.51 -4.16 9.06 0.01

Ecoregion 3 5.51 -4.16 9.06 0.01

Latitude 3 5.51 -4.15 9.07 0.01

Habitat 4 6.15 -2.82 10.04 0.01

Individual 4 5.74 -2.00 11.22 < 0.01

Subadult/adult Survival Individual 6 10.67 -5.99 0 0.98

(N = 32) Habitat 4 3.36 2.76 8.75 0.01

Null 2 -0.81 6.03 12.02 < 0.01

Year 6 3.11 6.08 12.07 < 0.01

Latitude 3 -0.58 8.02 14.01 < 0.01

Ecoregion 3 -0.76 8.38 14.37 < 0.01
