# Supplementary material for: Relative Importance of Climate Variables to Population Vital Rates: A Quantitative Synthesis for the Lesser Prairie-Chicken
Source: PLoS One. 2016 Sep 29;11(9):e0163585. doi: 10.1371/journal.pone.0163585 (PMC5042413; doi:10.1371/journal.pone.0163585)
Supplement: S2 Table — (DOCX) [file pone.0163585.s006.docx]

Table S2. AIC_c_ tables for the climate model set predicting variability in lesser prairie-chicken vital rates, where K is the number of parameters and N is the sample size (number of estimates).

**Vital Rate Model K Log Likelihood AIC_c_ Δ*_i_* *ω_i_***

Clutch Size Null 7 -35.28 89.43 0 0.54

(N = 31) Average Precipitation, 10 -30.22 91.44 2.01 0.20

Ecoregion Interaction

Drought 8 -34.81 92.17 2.74 0.16

Average Temperature 8 -35.26 93.06 3.63 0.10

Extreme Precipitation 9 -33.90 94.38 4.95 0.05

Extreme Temperature 9 -34.29 95.36 5.93 0.03

Average Precipitation 9 -34.59 95.75 6.33 0.03

Drought, Ecoregion 10 -34.38 99.77 10.34 < 0.01

Interaction

Average Temperature, 12 -31.31 103.95 14.52 < 0.01

Ecoregion Interaction

Extreme Precipitation, 12 -31.34 104.01 14.59 < 0.01

Ecoregion Interaction

Extreme Temperature, 12 -34.34 110.01 20.58 < 0.01

Ecoregion Interaction

Nest Success Null 4 11.35 -13.22 0 0.46

(N = 32) Drought 5 11.81 -11.31 1.91 0.18

Extreme Temperature 6 12.92 -10.47 2.74 0.12

Extreme Precipitation 6 12.62 -9.89 3.33 0.09

Average Precipitation 6 12.55 -9.75 3.47 0.08

Average Temperature 5 12.18 -9.01 4.21 0.06

Drought, Ecoregion 7 13.09 -7.52 5.70 0.02

Interaction

Average Precipitation, 9 13.45 -0.73 12.49 < 0.01

Ecoregion Interaction

Extreme Precipitation, 9 13.43 -0.68 12.53 < 0.01

Ecoregion Interaction

Extreme Temperature, 9 13.16 -0.13 13.09 < 0.01

Ecoregion Interaction

Average Temperature, 9 13.01 0.17 13.38 < 0.01

Ecoregion Interaction

Subadult/Adult Survival Null 4 8.59 -7.69 0 0.27

(N = 32) Extreme Precipitation 6 11.52 -7.68 0.01 0.27

Average Precipitation 6 10.70 -6.04 1.65 0.12

Average Temperature 6 10.68 -6.00 1.69 0.12

Drought 5 9.10 -5.90 1.79 0.11

Extreme Temperature 6 10.55 -5.75 1.94 0.10

Drought, Ecoregion 7 9.35 -0.04 7.65 0.01

Interaction

Extreme Temperature, 9 13.09 -0.00 7.69 0.01

Ecoregion Interaction

Extreme Precipitation, 9 11.69 2.80 10.49 < 0.01

Ecoregion Interaction

Average Temperature, 9 11.63 2.92 10.61 < 0.01

Ecoregion Interaction

Average Precipitation, 9 11.01 4.16 11.85 < 0.01

Ecoregion Interaction
